# Cross-Sectional Analysis of the Resistance of RC Members Subjected to Bending with/without Axial Force

**DOI:** 10.3390/ma15051957

**Published:** 2022-03-06

**Authors:** Marek Lechman

**Affiliations:** Instytut Techniki Budowlanej, ul. Filtrowa 1, 00-611 Warszawa, Poland; m.lechman@itb.pl; Tel.: +48-22-5796-117

**Keywords:** resistance, bending, axial force, reinforced concrete, composite, section models

## Abstract

This paper deals with the cross-sectional analysis of the resistance of RC members subjected to a bending moment with or without axial forces. To determine section resistance, the nonlinear material law for concrete in compression is assumed according to Eurocode 2, taking into account the effect of concrete softening. It adequately describes the concrete behavior of RC members up to failure. The idealized stress–strain relation for the reinforcing steel is assumed. For the ring cross-section subjected to bending with axial force and for areas weakened by an opening, normalized resistances have been derived by integrating corresponding equilibrium equations. They are presented in the form of interaction curves and compared with the results of testing conducted on RC eccentrically loaded columns. Furthermore, the ultimate normalized bending moment has been derived for the RC rectangle subjected to bending without axial force. It was applied to the cross-sectional analysis of steel and concrete composite beams consisting of the RC rectangular core located inside a reversed TT-welded profile. Comparative analysis indicated good agreements between the proposed section models and experimental data. The objective of the paper is the dimensioning optimization of the considered cross-sections with the fulfillment of structural safety requirements.

## 1. Introduction

For structural safety reasons, the load-bearing capacity (resistance) of any designed or existing structures should satisfy the conditions of ultimate limit states. In recent years, intensive developments and advanced applications of RC structural members with the increased resistances are observed in newly designed and erected tower buildings. The resistance of RC (reinforced concrete) members subjected to bending with or without axial force is undertaken both as a structural and a practical task. Such members are commonly encountered in engineering practices, e.g., reinforced concrete columns, tower-like structures, steel and concrete composite columns and beams. In the formulation accepted in this paper, the resistance of RC cross-section is determined by the occurrence of the ultimate strains occurring anywhere in that section, which means that it depends both on the material laws and the geometrical characteristics of the section. With respect to material laws, a simplified approach is the most often used on the basis of the rectangular stress distribution in concrete, represented among others by Knauff [1]. For the design of annular cross-sections, a parabola–rectangle diagram for concrete in compression is commonly assumed, which was introduced by Nieser and Engel [2] in German code DIN 1056 as well as in CICIND Model Code for Concrete Chimneys [3]. The dimensioning diagrams attached to these codes were developed on the assumption of the thin ring’s thickness and central layout of reinforcement. The parabolic–trapezoidal stress distribution for concrete in compression was proposed in turn by Hognestad [4] and applied in ACI Standard 307-08 [5]. A review of material laws for concrete and the experimental justification of formulae for the estimation of the complete stress–strain diagram of concrete were presented by Popovics [6,7]. For analysis of the resistance of noncircular cross-sections, deformation models were proposed by Lechman [8] on the basis of the parabola–rectangle stress distribution. Moreover, the author developed an algorithm for determining the analyzed resistance that takes into account the effect of concrete softening [9,10]. Among the structural members that underwent eccentric loading, reinforced concrete columns are the most encountered ones in the engineering practice. Majewski et al. [11] and Rodrigues et al. [12] presented the results of FE modeling of failure behavior of RC eccentrically loaded columns. A computational method with verification by a series of tests for predicting the behavior of RC columns subjected to axial force and biaxial bending was proposed by Kim and Lee [13]. The eccentricity of the applied axial load causes significant variation in the failure load and mode. The load-bearing capacity of RC columns under eccentric compression was investigated among others by Lloyd et al. [14]; Chruściel [15]; and Trapko et al. [16]. Strengthening and repairing techniques of RC columns by means of CFRP, FRP and GFRP composites currently attract the attention of many researchers and engineers. The results of the performed investigations in this range are reported among others by Campione et al. [17]; Maaddawy et al. [18]; Elwan and Rashed [19]; Sadeghian et al. [20]; Eid and Paultre [21]; Wu and Jiang [22]; and Quiertant and Clement [23]. When increased column resistances are needed, concrete-filled steel tubular columns (CFST) or concrete-filled double skin steel tubular columns (CFDST) may form effective options [24,25,26,27]. For the given normal force *N* and bending moment *M* and when the nonlinear material law for concrete is assumed, the task consists in the determining the unknown section strains. In this case, the problem is described mathematically by a set of two equations that are highly nonlinear and difficult to be solved. Therefore, a numerical optimization strategy must be employed [28].

Newly designed floor slab systems are currently the subject of particular interest of designers because they provide a flat lower surface of finished floor slab. These systems consist of steel and concrete composite beams that are structurally connected with prefabricated or cast in situ slabs [29,30,31,32]. The results of FEM modeling of their failure behavior revealed that significant differences in the ultimate bending moments occur compared to the bending test results. Therefore, the cross-sectional analysis was employed to solve this problem. The related issues were presented in other research papers concerning the resistance of RC buildings subjected to vertical earthquake or blast loads [33,34], as well as reporting the examination results of prestressed concrete beams and so-called concrete structures with symmetries [35,36,37]. The current state of knowledge showed that despite the variety of calculation and experimental methods concerning the paper topic, there are no analytical solutions for the section resistance based on the nonlinear material laws and taking into account the effect of concrete softening. In this contribution, an analytical solution for the ring section resistance is being formed step-by-step and presented in the form of the actual carrying capacity curves in axial force–bending moment, which satisfy the stability conditions by Drucker [38]. Moreover, the ultimate normalized bending moment is derived for the RC rectangle subjected to bending without axial force and applied for determining the resistance of steel and concrete composite beams. Both proposed section models were verified by experimental results to confirm their suitability in the engineering practice.

In the presented considerations, two basic assumptions are made in which plane cross-sections remain in plane and the tensile strength of concrete is neglected.

According to Eurocode 2, the stress–strain relation for concrete *σ_c_*-*ε_c_* in compression for short term uniaxial loading is recommended for nonlinear structural analysis as follows [39].

(1) $$\sigma_{c}=\frac{k\eta -\eta^{2}}{1+(k-2)\eta }f_{cm}$$

For both reinforcing and profile steels, the linear elastic–ideal plastic model is applied.

## 2. Ring Cross-Section Subjected to Bending with Axial Force

### 2.1. Derivation of Analytical Formulae for the Resistance

**Figure 1:**
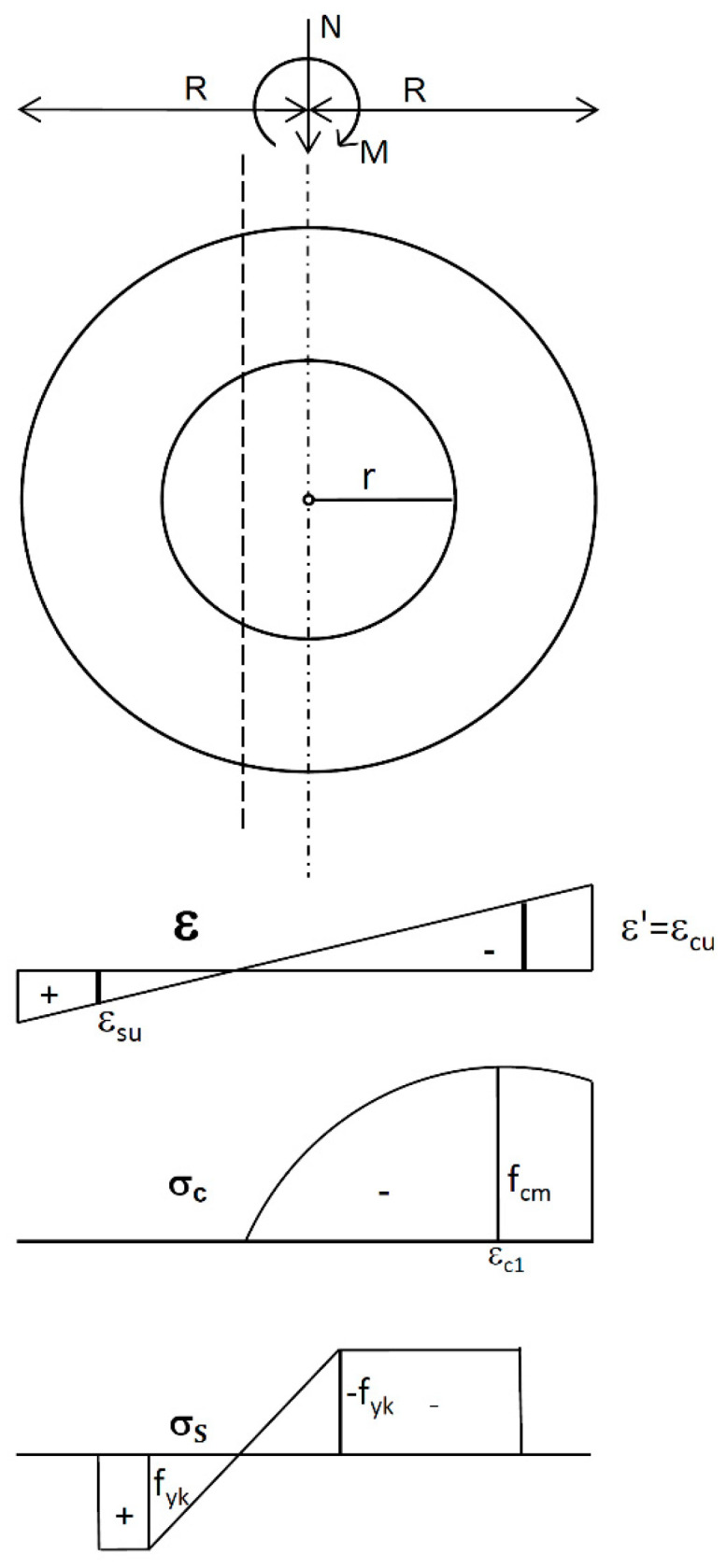
Representation of the RC ring cross-section; distribution of strains *ε*; stresses in concrete *σ_c_*; and stresses in reinforcement *σ_s_*.

- The RC ring cross-section of outer radius *R*, inner radius *r* and thickness *t* = *R* − *r* is subjected to axial force *N* and bending moment *M* (Figure 1). The section may be unreinforced or reinforced with the reinforcing steel spaced at one or two layers, which can be replaced by a continuous ring of equivalent area located on the reference circumference of radius *r_s_*, *r* ≤ *r_s_* ≤ *R*. When *r_s_* = *R* or *r_s_* = *r*, this reinforcement is treated as an “external reinforcement”.
- For the section under combined compression and bending, the relations for strains in concrete *ε_c_* (‰) and in reinforcing steel *ε_s_* (‰) are given by the following.

(2) $$\varepsilon_{c}=(\cos\phi -\cos\alpha )\varepsilon_{\alpha }^{\prime };$$

(3) $$\varepsilon_{s}=(\rho \cos\phi -\cos\alpha )\varepsilon_{\alpha }^{\prime }.$$

All angles are measured from the compressive to the tensile zone. The sectional equilibrium equation of the axial forces is described as follows.

(4) $$\int_{A_{c}}^{}\sigma_{c}dA_{c}+\int_{A_{s}}^{}\sigma_{s}dA_{s}+N\;=\;0$$

The sectional equilibrium equation of the bending moments about the symmetry axis of the section is expressed as follows.

(5) $$\int_{A_{c}}^{}\sigma_{c}\;r_{m}\cos\phi \;dA_{c}+\int_{A_{s}}^{}\sigma_{s}\;r_{s}\cos\phi \;dA_{s}-M\;=\;0$$

Taking into account physical and geometrical relationships (1)–(3) in equilibrium Equations (4) and (5), the problem results in a purely mathematical task consisting in searching the indefinite integrals of the following functions of variable *ϕ*:

(6) $$f_{N}(\phi )=\frac{k\;k_{2}(\cos\phi \;-\;k_{1})-\;k_{2}^{2}{\left(\cos\phi -\;k_{1}\right)}^{2}}{1+(k-2)\;k_{2}(\cos\phi -\;k_{1})};$$

(7) $$f_{M}(\phi )=\frac{k\;k_{2}(\cos\phi \;-\;k_{1})-\;k_{2}^{2}{\left(\cos\phi -\;k_{1}\right)}^{2}}{1+(k-2)\;k_{2}(\cos\phi -\;k_{1})}\;\cos\phi ,$$

where the following is the case.

(8) $$k_{1}=\cos\alpha ;\;k_{2}=\varepsilon_{\alpha }^{\prime }\;/\varepsilon_{c1}.$$

Upon transformation, Equations (6) and (7) result in the following:

(9) $$f_{N}(\phi )=(k_{2}/(k-2))[\sin\phi -(k_{1}+W_{2})+2\;W_{1\;}\;W_{2}\frac{1}{\cos\phi +b})];$$

(10) $$f_{M}(\phi )=(k_{2}/(k-2))\;[0.5\;(0.5\;\sin2\phi +\phi )+W_{1}\;W_{2}\;\phi -k_{1\;}\;W_{2}\sin\phi -\;2\;W_{1}\;W_{2}\;b/(\sqrt{b^{2}-1}\;)\frac{1}{\cos\phi +b}],$$

where *W*_1_ = 1/(*k* − 2)*k*_2_; *W*_2_ = *W*_1_ + *k*/*k*_2_; and *b* = *W*_1_ − *k*_1_.

An indefinite integral of function 1/(cos*ϕ* + *b*) occurring in Equations (9) and (10) has been found as follows.

(11) $$\int \frac{d\phi }{\cos\phi +b}\;=\;\frac{2}{\sqrt{b^{2}-1}}\;arctg\frac{(b-1)tg0.5\phi }{\sqrt{b^{2}-1}}\;\mathrm{for}\;b^{2}>1;$$

(12) $$\int \frac{d\phi }{\cos\phi +b}=\frac{1}{\sqrt{1-b^{2}}}\;\ln|\frac{(1-b)tg0.5\phi +\sqrt{1-b^{2}}}{(1-b)tg0.5\phi \;-\sqrt{1-b^{2}}}|\;\mathrm{for}\;b^{2}<1.$$

Having calculated the definite integrals of Equations (9)–(12), normalized ultimate resistances *n_Rm_* and *m_Rm_* are obtained in the following final form:

(13) $$\begin{array}{l} -n_{Rm}=(k_{2}/(k-2))\left\{\sin\alpha -(k_{1}+W_{2})\alpha +2\;W_{1\;}\;W_{2}/(\sqrt{b^{2}-1}\;)\;\mathrm{arctg}(\frac{\left(b-1)\mathrm{tg}(0.5\alpha \right)}{\sqrt{b^{2}-1}})\right\}+\\ \;\;\;\;\;\;\;\;\;\;\;\;\;\;\;\;\;\;\;\;\;\;\;\;\;\;\;\;\;\;\;+\mu_{}\frac{f_{yk}}{f_{cm}}\left\{\begin{array}{l} -\alpha_{1}+\frac{\varepsilon_{\alpha }^{\prime }}{\varepsilon_{ss}}(\sin\alpha_{2}-\sin\alpha_{1}-\cos\alpha \;(\alpha_{2}-\alpha_{1}))\\ \left[\pi -\alpha_{2}+\varepsilon_{ss}\;(\pi -\alpha_{2})\right] \end{array}\right\} \end{array}$$

(14) $$\begin{array}{l} m_{Rm}=(k_{2}/(k-2))\left\{\begin{array}{l} 0.5\;(0.5\;\sin2\alpha +\alpha )+W_{1}\;W_{2}\;\alpha -k_{1\;}\;W_{2}\sin\alpha -\;2\;W_{1}\;W_{2}\;b/(\sqrt{b^{2}-1}\;)\;\\ \mathrm{arctg}(\frac{\left(b-1)\mathrm{tg}(0.5\alpha \right)}{\sqrt{b^{2}-1}}) \end{array}\right\}\\ \;\;\;\;\;\;\;\;\;\;\;\;\;\;\;\;\;+\;\mu_{}\frac{f_{yk}}{f_{cm}}\left\{\begin{array}{l} \left[-\sin\alpha_{1}+\varepsilon_{ss}\;\sin\alpha_{1})\right]+\\ \frac{\varepsilon_{\alpha }^{\prime }}{\varepsilon_{ss}}(0.5(\alpha_{2}-\alpha_{1})+0.5(\sin2\alpha_{2}-\sin2\alpha_{1}-\cos\alpha \;(\sin\alpha_{2}-\sin\alpha_{1}))+\\ \left[-\sin\alpha_{2}+\varepsilon_{ss}\;\sin\alpha_{2})\right] \end{array}\right\} \end{array}$$

where the following is the case.

*N_Rm_* = *N*/(π *d_m_ t f_cm_*); *m_Rm_* = *M*/(π *d_m_*^2^
*t f_cm_*), (15)

Here, *μ* is the reinforcement ratio; and *α*_1_ and *α*_2_ are angles determining the depth of the plastified zones of steel in compression and in tension, respectively.

**Figure 2:**
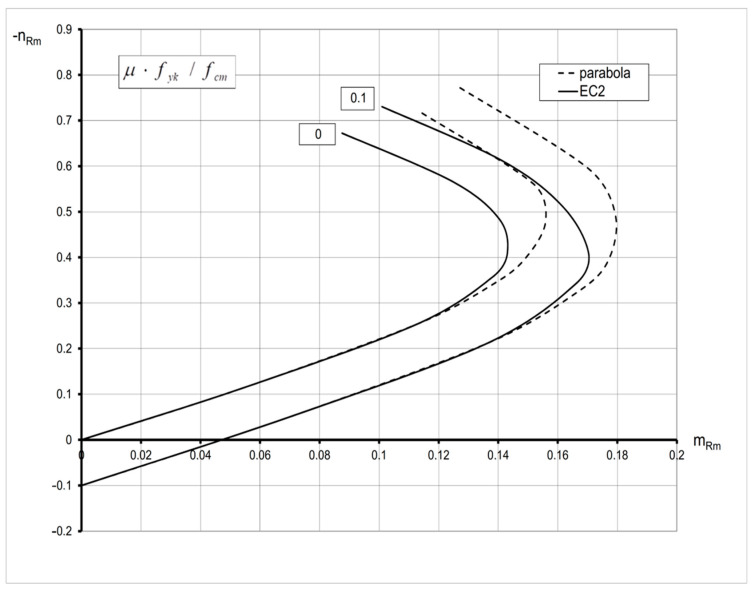
Interaction curves *n_Rm_*-*m_Rm_* based on nonlinear relation *σ_c_*-*ε_c_* (EC2) versus those based on the parabola–rectangle material law (parabola) for the ring section subjected to bending with axial compressive force (*ε_cu_* = −3.5‰).

The graphic interpretations of Equations (13)–(15) are the interaction diagrams with normalized resistances *n_Rm_*-*m_Rm_* valid for the case, when both compressive and tensile strains occur in the analyzed section characterized by the following: the limiting value *ε_cu_ =* −3.5‰; the concrete grade C20/25 (*f_cm_* = 28 MPa); *f_yk_* = 500 MPa; *E_cm_* = 30 GPa; substitute reinforcement ratio *μ f_yk_/f_cm_* = 0; 0.1; 0 ≤ *ε_s_* ≤ 10‰ (Figure 2). These curves denoted by the solid line (EC2) are compared with those based on the parabola–rectangle diagram (the dashed line, parabola). It is apparent that the coordinates of curves denoted by EC2 are lower than for the parabola due to the effect of concrete softening.

In a similar manner, the corresponding formulae are obtained for the section that is entirely in compression. The relevant relationship for strains is given by the following.

(16) $$\varepsilon =0.5\;(\varepsilon_{2}-\varepsilon_{1})\;(1-\cos\phi )+\varepsilon_{1}.$$

The formulae determining cross-sectional forces *n_Rm_* and *m_Rm_* are described in the following form:

(17) $$\begin{array}{l} -n_{Rm}=(k_{2}/(k-2))\pi \{-(k_{1}+W_{2})+\;W_{1\;}\;W_{2}/(\sqrt{b^{2}-1}\;)\}+\\ \left(\mu \;f_{yk}/f_{cm})\{-\alpha_{1}+1/\varepsilon_{ss}[(k_{1}+k_{2})(\alpha_{2}-\alpha_{1})+k_{2}(\sin\alpha_{1}-\sin\alpha_{2})+\pi -\alpha_{2}]\right\} \end{array}$$

(18) $$\begin{array}{l} \;\;\;\;\;\;\;\;\;\;\;\;\;\;\;\;\;\;\;\;\;\;\;\;\;\;\;\;\;\;\;\;\;\;\;\;\;\;\;\;m_{Rm}=-(k_{2}(k-2))\pi \{\;0.5\;+W_{1}\;W_{2}\;-\;2\;W_{1}\;W_{2}\;b/(\sqrt{b^{2}-1}\;)\}+\\ \left(\mu \;f_{yk}/f_{cm})\{-\sin\alpha_{1}+1/\varepsilon_{ss}[(k_{1}+k_{2})(\sin\alpha_{2}-\sin\alpha_{1})-0.5(\alpha_{2}-\alpha_{1}+\sin2\alpha_{2}-\sin2\alpha_{1})-\sin\alpha_{2}]\right\} \end{array}$$

where *k*_1_ = 2*ε*_1_/(*ε*_2_ − *ε*_1_) + 1; and *k*_2_ = −0.5 (*ε*_2_ − *ε*_1_)/*ε_c_*_1_ shall be substituted.

**Figure 3:**
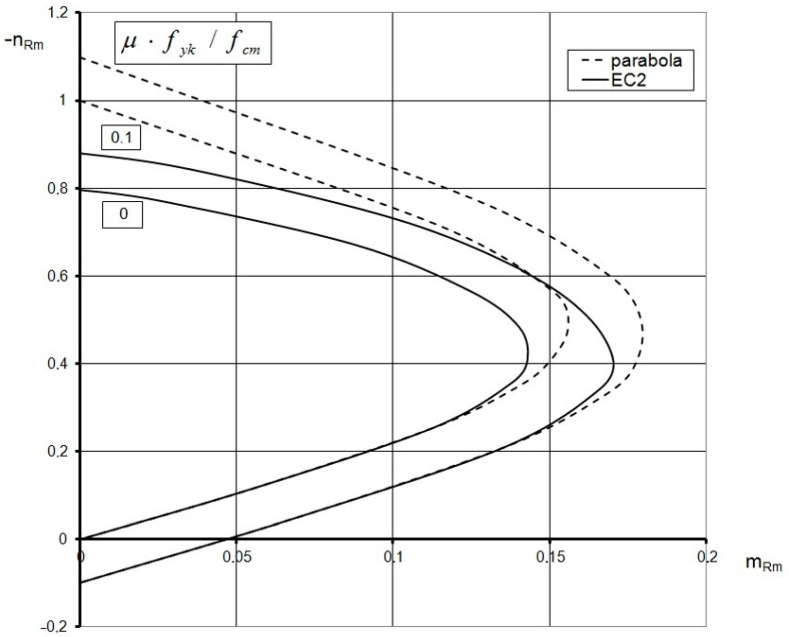
Interaction curves *n_Rm_*-*m_Rm_* based on nonlinear relation *σ*_c_-*ε*_c_ (EC2) versus those based on the parabola–rectangle material law for concrete (parabola), extended for the ring cross-section that is entirely in compression (*ε_cu_* = −3.5‰; *m_Rm_* ≥ 0).

**Figure 4:**
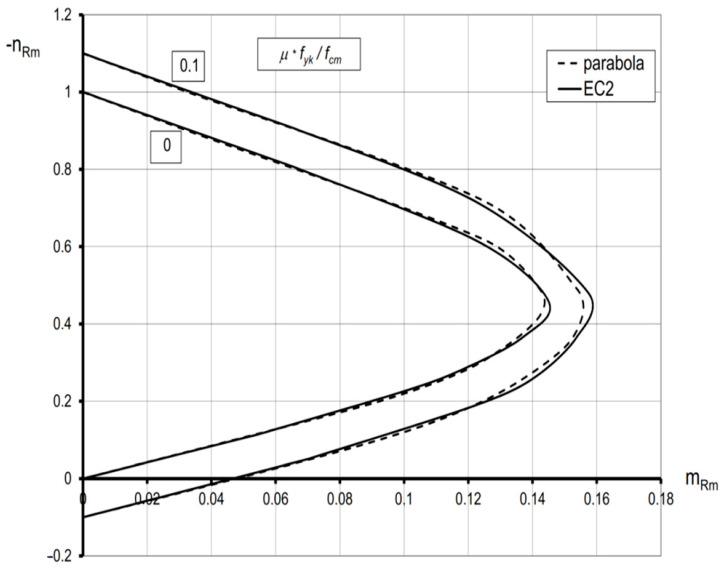
Comparison of the solution based on nonlinear relation *σ_c_*-*ε_c_* (EC2) with that based on the parabola–rectangle material law for the ring cross-section (parabola) and the limiting value *ε_cu_ = ε_c_*_1_ = −2‰.

For the section that is entirely in compression, when |*ε_cu_* | > |*ε_c1_* |, Equations (17) and (18) describe the ring cross-section at the stage of concrete instability (at failure). This means that Drucker’s stability postulates may not be satisfied [38,40]. Therefore, the obtained curves cannot be, in general, regarded as the carrying capacity curves. This is the case presented in [9]. Figure 3 presents interaction curves (EC2) resulting in the relation (1) versus those derived on the basis of the parabola–rectangle (parabola) extended to the ring cross-section that is entirely in compression (*ε_c_*_1_ = −2.0 ‰; *ε_cu_ =* −3.5‰), with a limitation to the values *m_Rm_* ≥ 0. All curves in Figure 2 and Figure 3 satisfy the condition of convexity according to Drucker’s postulate. In particular, for *ε_cu_* = *ε_c_*_1_ = −2‰, the stability condition by Drucker is satisfied for any values of *ε_c_* and *ε_s_*. Thus, the obtained curves based on Equations (13)–(15) and Equations (17) and (18) can be regarded as the actual carrying capacity curves accepted in design codes. It is worth highlighting that these interaction diagrams (EC2) are very close to those based on the parabola–rectangle (parabola) (Figure 4).

**Figure 5:**
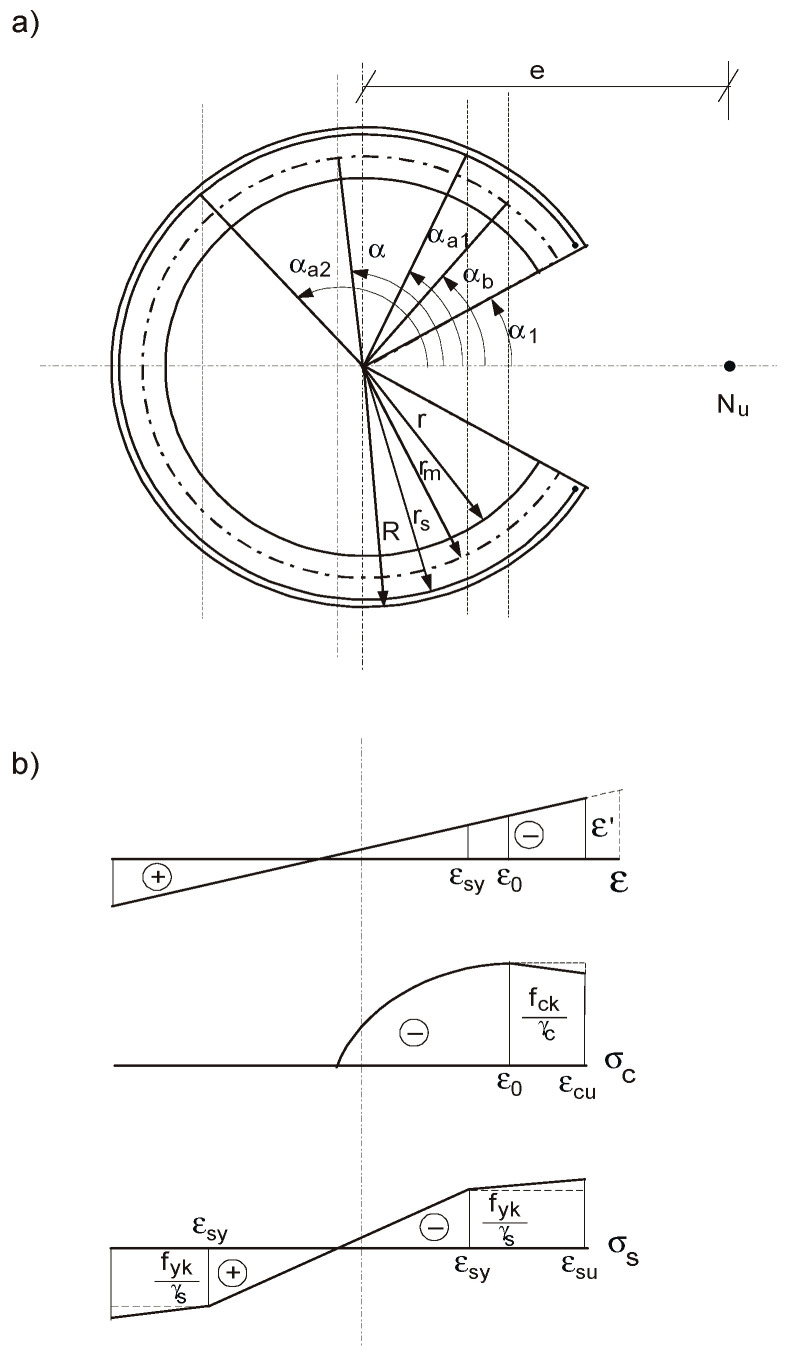
(**a**) Representation of the RC ring cross-section weakened by one opening; (**b**) distribution of strains *ε*, stresses in concrete *σ_c_* and stresses in reinforcing steel *σ_s_*_._

As the next section model, the ring cross-section weakened by one opening is considered and is subjected to ultimate axial force *N_u_* and bending moment *M_u_* = *N_u_* *⋅ e* (Figure 5). The stress distributions in concrete and reinforcing steel are described by design values *f_cd_* = *f_ck_/γ_c_* and *f_yd_* = *f_yk_/γ_s_*, while the size of opening is denoted by angle *α*_1_. Following the above outlined algorithm, normalized ultimate resistances *n_u_* and *m_u_* are derived for this section in the following form:

(19) $$\begin{array}{c} +\mu \frac{f_{yd}}{f_{cd}}[\alpha_{1}-\alpha_{a1}+\frac{\varepsilon_{\alpha }^{\prime }}{\varepsilon_{ss}}(\sin\alpha_{a2}-\sin\alpha_{a1}-\cos\alpha \;(\alpha_{a2}-\alpha_{a1}))+\pi -\alpha_{a2}];\\ -n_{u}=(k_{2}/(k-2))\left\{\begin{array}{l} \sin\alpha -\sin\alpha_{1}-(k_{1}+W_{2})(\alpha -\alpha_{1})+2\;W_{1\;}\;W_{2}/(\sqrt{b^{2}-1}\;)\;[\mathrm{arctg}(\frac{\left(b-1)\mathrm{tg}(0.5\alpha \right)}{\sqrt{b^{2}-1}})+\\ -\mathrm{arctg}(\frac{\left(b-1)\mathrm{tg}(0.5\alpha_{1}\right)}{\sqrt{b^{2}-1}})] \end{array}\right\} \end{array}$$

(20) $$\begin{array}{c} m_{u}=(k_{2}/(k-2))\left\{\begin{array}{l} 0.5\;[0.5\;(\sin2\alpha -\sin2\alpha_{1})+\alpha -\alpha_{1}]+W_{1}\;W_{2}\;(\alpha -\alpha_{1})-k_{1\;}\;W_{2}(\sin\alpha -\sin\alpha_{1})+\;\;\\ -2\;W_{1}\;W_{2}\;b/(\sqrt{b^{2}-1}\;)[\mathrm{arctg}(\frac{\left(b-1)\mathrm{tg}(0.5\alpha \right)}{\sqrt{b^{2}-1}})-\mathrm{arctg}(\frac{\left(b-1)\mathrm{tg}(0.5\alpha_{1}\right)}{\sqrt{b^{2}-1}})] \end{array}\right\}\\ +\mu \frac{f_{yd}}{f_{cd}}[\sin\alpha_{1}-\sin\alpha_{a1}\;+\frac{\varepsilon_{\alpha }^{\prime }}{\varepsilon_{ss}}(0.5(\alpha_{a2}-\alpha_{a1})+0.5(\sin2\alpha_{a2}-\sin2\alpha_{a1}-\cos\alpha \;(\sin\alpha_{a2}-\sin\alpha_{a1}))\\ -\sin\alpha_{a2}] \end{array}$$

where *α_a_*_1_ and *α_a_*_2_ are the angles determining the depth of the plastified zones of steel in compression and in tension, respectively.

The section model is frequently used in the structural design, e.g., flue opening in chimneys, windows in tower walls [8]. In a similar manner, the corresponding interaction diagrams can be constructed for other section shapes.

### 2.2. Experimental Verification with Discussion of the Results

**Figure 6:**
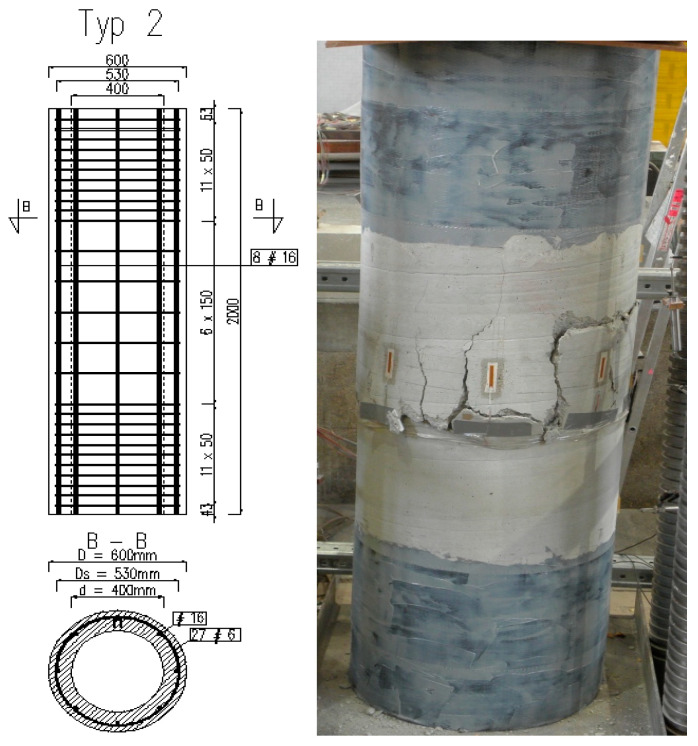
The specimen and the failure mode of the column under eccentric loading.

**Figure 7:**
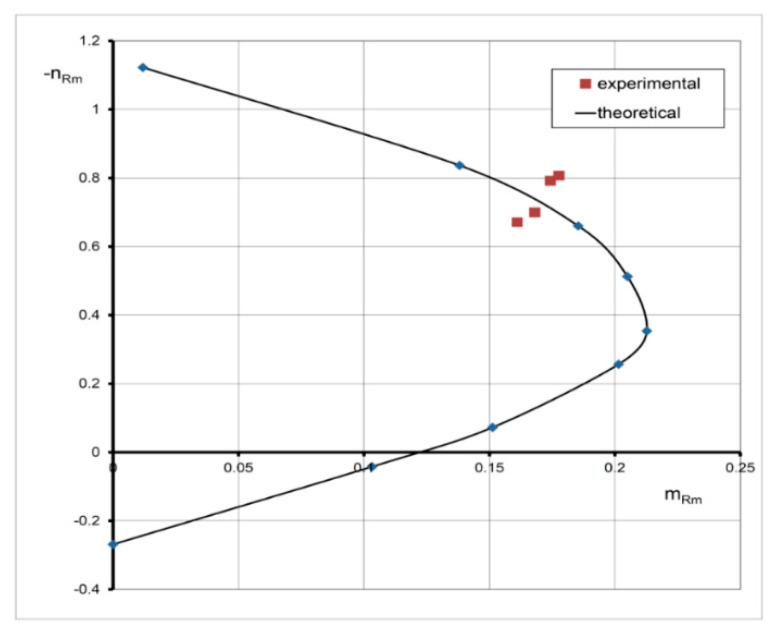
Comparison of theoretical and experimental data.

Full-scale tests were carried out on four RC designed columns eccentrically loaded, with annular cross-section, denoted by Typ 2 [15]. The outer radius of all columns was *R* = 0.3 m, the inner one was *r* = 0.2 m and the height was *h* = 2.0 m. The mean compressive strength of the column concrete was determined as *f_cm_* = 20 MPa; *E_cm_* = 27 GPa; and *ε_c_*_1_ = −1.8‰ by strength test according to Polish Standard PN-EN 12390-3: 2011 [15]. The reinforcement of the columns consisted of longitudinal bars ∅16 mm made of steel B500C (*f_yk_* = 500 MPa) and its percentage was *μ* = 1.024%. The properties of the steel rebars were established in turn by tensile test according to Polish Standard PN-EN ISO 6892-1:2010, [15]. The columns were strengthened in the both support zones by CFRP mats at the length of 0.5 m. The tested specimens and the experimental setup are exhibited in Figure 6. In each load step, the strains were measured in the middle section using strain gauges located along its circumference up to failure. The test results of the examined columns are collected in Table 1. The failure of the columns manifested itself by crushing the concrete and yielding the longitudinal reinforcing steel for all tested members (Figure 6). The above-described test results have been compared with the cross-section model presented in Section 2.1. with the substitute reinforcement ratio of *μ f_yk_/f_cm_* = 0.2688. Using the derived Equations (13)–(18), the interaction chart *n_Rm_*-*m_Rm_* has been plotted. The effect of confinement of the column (stirrups) was not analyzed. The comparisons presented in Figure 7 illustrates a good convergence between the section model and the values of failure loads. The occurring differences between the analytical and the experimental results are caused by measurement uncertainty (strains, eccentricity) as well as by ignoring transverse reinforcement in the section model.

## 3. RC Rectangle Subjected to Bending without Axial Force

### 3.1. Derivation of the Ultimate Bending Moment

**Figure 8:**
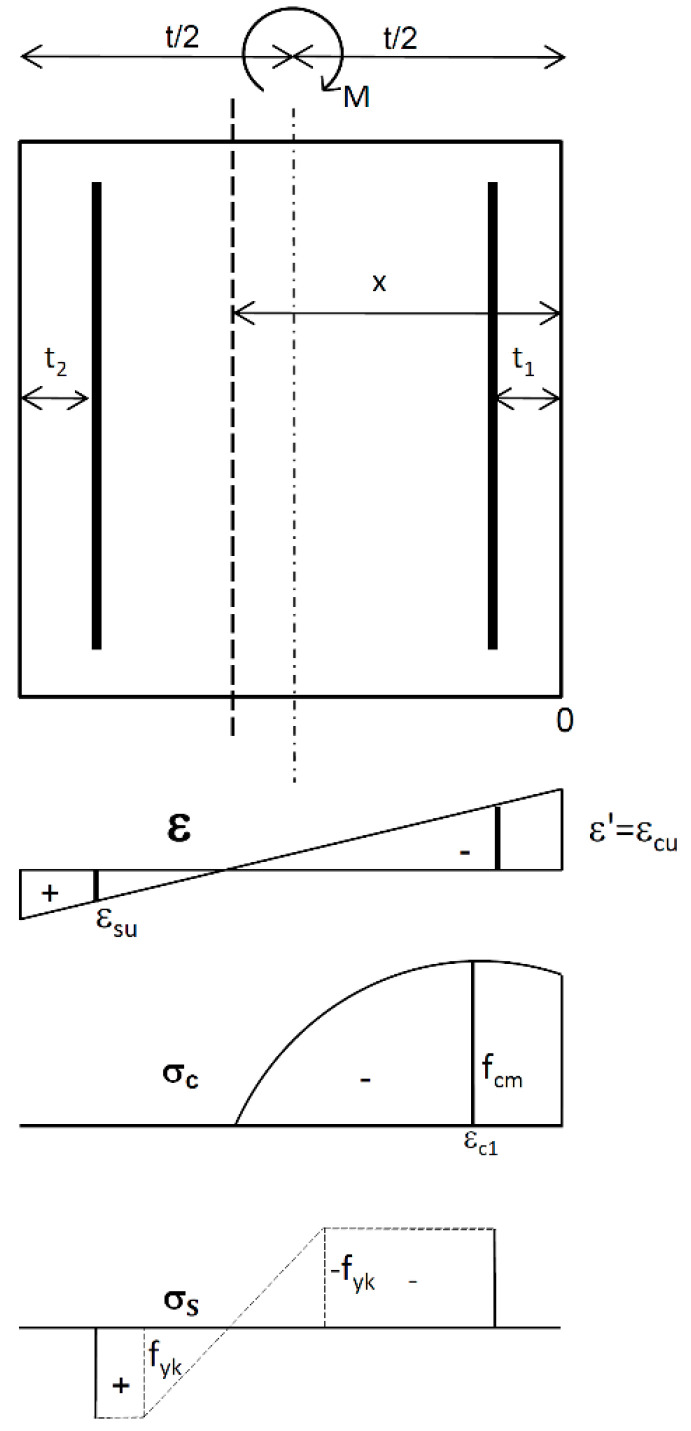
The RC rectangle; distribution of strain *ε*, stresses in concrete *σ_c_* and stresses in steel *σ_s_*.

The RC rectangle of the height *t* and the width *b* is subjected to bending moment *M* without axial force (Figure 8).

The relation for strains can be expressed in the following form:

(21) $$\varepsilon =(1-\frac{\xi^{\prime }}{\xi })\varepsilon^{\prime },$$

where *ξ′ = x*’/*t* and *ξ* = *x*/*t* is the dimensionless coordinate of any point of the rectangle and the dimensionless coordinate describing the location of neutral axis.

The equilibrium equation of the bending moments about the horizontal axis of the RC rectangle is described as follows.

(22) $$\int_{0}^{x}\sigma_{c}\;(0.5t-x^{\prime }\;)dA_{c}+\sigma_{s1}\;F_{a1}\;(0.5t\;-t_{1})+\sigma_{s2}\;F_{a2}\;(0.5t-t_{2})-M\;=\;0$$

As a result of integrating Equation (22), the normalized ultimate bending moment *m_Rm_* is obtained in the following final form.

(23) $$\begin{array}{l} m_{Rm}=(1/(k-2))\left\{\begin{array}{l} 0.5(W_{1}+(1/(k-2)))\xi +0.5\left[-W_{1}+0.5k_{2}-((1/(k-2))\right]\xi^{2}+\\ -(1/3)k_{2}\xi^{3}-(W_{{}_{2}}/((k-2)W_{3}))\left[0.5\ln W+\xi -(W/W_{3})\ln W\right] \end{array}\right\}\\ +\mu_{1}\frac{f_{yk}}{f_{cm}}(0.5-\xi_{1})\left\{-\delta_{k1}+\delta_{k1+1}\frac{\varepsilon^{\prime }}{\varepsilon_{ss}}(1-\frac{\xi_{1}}{\xi })\right\}+\mu_{2}\frac{f_{yk}}{f_{cm}}(0.5-\xi_{2})\left\{\delta_{k2}+\delta_{k2+1}\frac{\varepsilon^{\prime }}{\varepsilon_{ss}}(1-\frac{1-\xi_{2}}{\xi })\right\} \end{array}$$

(24) $$m_{Rm}=\frac{M_{Rm}}{bt^{2}f_{cm}}$$

(25) $$\xi =\frac{(1-\xi_{1})\varepsilon_{cu1}}{-\varepsilon_{su}+\varepsilon_{cu1}};\;\varepsilon =\varepsilon_{uc1};-(\frac{1}{\xi }-1)\varepsilon^{,}=\varepsilon_{su};$$

*k*_2_ = *ε′*/(*ε_c_*_1_ *ξ*); *W*_1_ = *k* − *k*_2_*ξ*; *W*_2_ = *k*(*k* − 2) + 1; *W*_3_ = (*k* − 2)*k*_2_; *W* = 1 + (*k* − 2)*k*_2_*ξ*; *δ_k_* = 0.5((−1)*^k^* + 1). (26)

### 3.2. The Resistance of Composite Steel and Concrete Beams Versus Test Results

**Figure 9:**
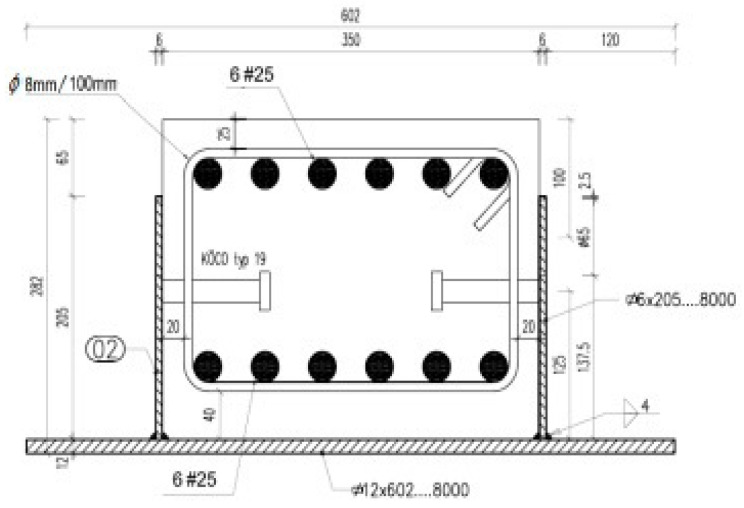
The cross-section of the composite beam under consideration.

The presented section model can be applied for determining the resistance of composite steel and concrete beams, named BH beams, subjected to bending. The considered beams consist of a reinforced (RC) rectangular core placed inside a reversed TT-welded profile, as shown in Figure 9.

**Figure 10:**
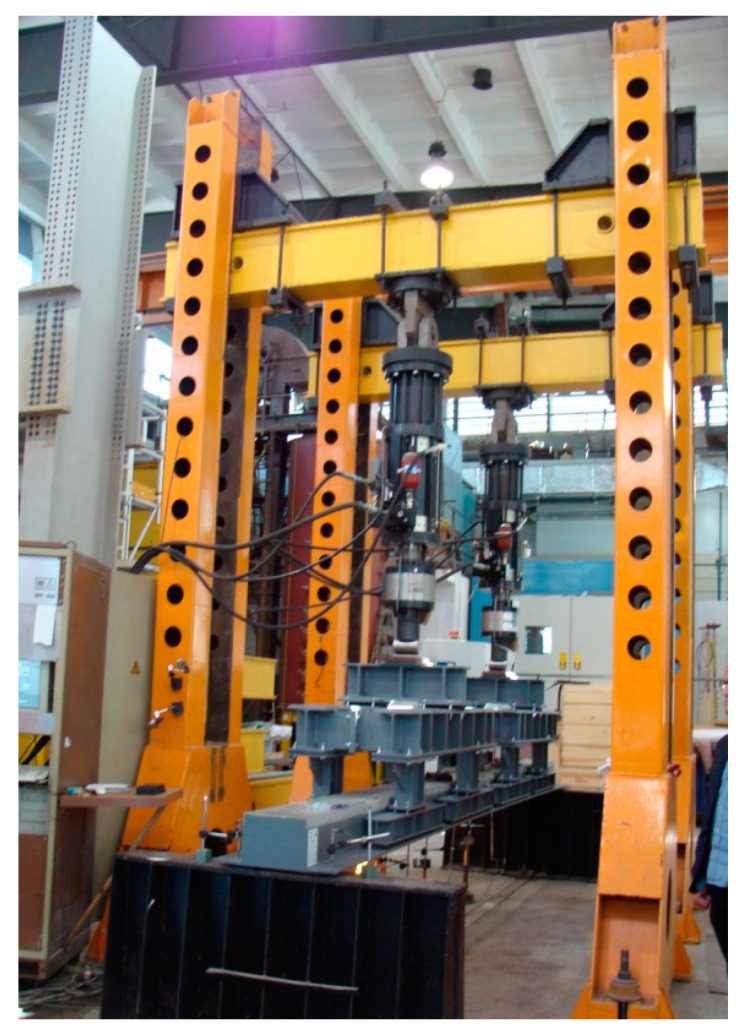
Setup of the bending tests.

Ultimate bending moment *M_HRm_* determining the resistance of the considered BH beam is derived in terms of strains upon integrating the equilibrium equation of the bending moments about the horizontal axis of the RC core rectangle [28]. In this derivation, the reversed TT-welded profile is treated as the external reinforcement with respect to the RC rectangular core. To compare the obtained analytical solution with experimental results, four-point bending tests were conducted on three separated BH beams with the length of *L* = 7.88 m. They were made of concrete with a mean compressive strength of *f_cm_* = 68 MPa, which was determined by a strength test according to Polish Standard PN-EN 12390-3:2011 (concrete grade C 60/75; *E_cm_* = 39 GPa). For the RC rectangle with the cross-section of 0.27 m × 0.35 m, the reinforcing steel with *f_yk_* = 500 MPa and the profile steel with *f_Hyk_* = 460 MPa were used. The properties of the steels were established in turn by tensile tests according to Polish Standard PN-EN ISO 6892-1:2010. The setup of the tests is exhibited in Figure 10. The range of the tests included determining failure loads and the relevant strains. In each load step, the strains in concrete *ε_c_*, in reinforcing steels in compression *ε_s1_* and in tension *ε_s_*_2_, as well as in the lower flange of profile steel *ε_Hf_*, were measured in the middle section of the BH beam.

**Figure 11:**
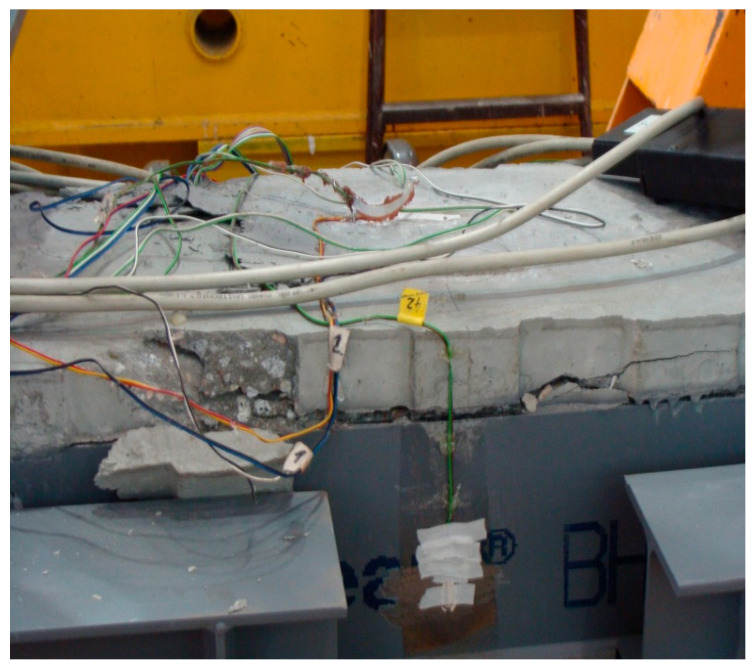
The characteristic failure mode of the BH beams.

The failures of all BH beams manifested themselves by concrete crushing (Figure 11). Table 2 summarizes the values of strains, failure bending moments *M_u_* and resistances *M_HRm_*. The compressive strains in concrete reached ultimate values. The value of *ε_s_*_1_ = −2.89‰ indicates that the plastic strains in the rebars in compression may have occurred. The values of *M_HRm_* have been calculated in accordance with derived Equations (23)–(26). It is worth noting that they are close to failure bending moments *M_u_* (relative differences 4.8–6.5%). This confirms very good agreements between resistances *M_HRm_* and the test results in ultimate bending moments *M_u_*.

## 4. Conclusions

The complete analytical solution has been found for the resistance of RC ring cross-sections subjected to bending with axial force, based on the nonlinear material law for concrete and taking into account the effect of concrete softening. It applies both to designed and existing members and structures. In this respect, it can be regarded as a valuable one in the theory of reinforced concrete:

1. The obtained solutions are presented in the form of interaction diagrams that satisfy the conditions of convexity in accordance with Drucker’s postulates. This means that they can be regarded as the actual carrying capacity curves.
2. The proposed ring section models seem to have a wider application field than the previous ones, due to the assumptions of a noncentral layout of reinforcement and wall-edge strains. As a result, they are suitable for ring cross-sections with both the thin and moderate thicknesses. Furthermore, they can be easily adopted when structure strengthening is required by means of externally bonded CFRP, FRP or GFRP composites as well as for determining the resistance of composite steel and concrete columns.
3. Using this approach, the similar formulae can be derived for other sections commonly encountered in the engineering practice, for example, a rectangle and the ones weakened by openings.
4. It was proved that the computational results conform to those obtained by testing on RC eccentrically loaded columns.
5. The analytical solution was developed for the resistance of RC rectangle subjected to bending without axial forces to determine ultimate bending moment *M_HRm_* of the composite steel and concrete beams.
6. The comparisons made between the computational and test results of BH beams showed good agreements in ultimate bending moments.
7. The above-developed models enabled the analysis of the behavior of RC members in the postcritical phase.
8. They have been implemented in Excel to provide a useful tool for the dimensioning optimization of RC-designed members and structures that may result in a reduction in material consumption and a lesser impact on the environment.
9. Further experimental work is needed concerning the postcritical behavior of RC members.

## Figures and Tables

**Table 1:** Test results of the eccentrically loaded columns.

Load Eccentricity (cm)	Failure Load (kN)	Ultimate Compressive Strain in Concrete (‰)
11	2490	2.6
12	2110	2.5
12	2200	2.5
11	2535	3.4

The mean value *ε_cu_* = 2.8‰.

**Table 2:** Bending test results of the BH beams.

Concrete	Reinforcement in Compression	Reinforcement in Tension	Profile Steel	Failure Moment	Resistance
strain *ε_c_* (‰)	strain *ε_s_*_1_ (‰)	strain *ε_s_*_2_ (‰)	strain *ε_Hf_* (‰)	*M_u_* (kNm)	*M_HRm_* (kNm)
−3.21	−2.89	1.15	2.22	902.8	847.7
−3.04	−2.13	1.13	2.25	883	842.4
−2.90	−2.0	1.25	2.28	910.7	854.8

## Data Availability

Not applicable.

## Nomenclature

*f_cd_* = *f_ck_/γ_c_*	design strength of concrete in compression
*f_ck_*	characteristic strength of concrete in compression
*γ_c_*	partial safety factor for concrete
*f_cm_*	mean compressive strength of concrete
*f_yk_*	yield stress of reinforcing steel
*f_yd_* = *f_yk_/γ_s_*	design yield stress of reinforcing steel
*γ_s_*	partial safety factor for steel
*f_Hyk_*	yield stress of profile steel
*E_cm_*	secant modulus of elasticity of concrete
*E_s_*	modulus of elasticity of steel (profile steel)
*ε_c_*_1_	strain at peak stress on the *σ_c_*_-_*ε_c_* diagram
*ε_cu_*_1_, *ε_cu_*	ultimate strain for concrete
*ε*_1_, *ε*_2_	maximum and minimum compressive strains, respectively
*ε_su_*	ultimate strain for reinforcing steel
*ε_Hf_*	strain in the lower flange of profile steel
*e*	eccentricity
*σ* *_c_, ε_c_*	stress, strain in concrete
*σ_s_, ε_s_*	stress, strain in reinforcing steel
*N*	axial force
*M*	bending moment
*N_u_, M_u_*	ultimate axial force and ultimate bending moment, respectively
*n_Rm_, n_u_*	normalized ultimate axial force
*m_Rm_, m_u_*	normalized ultimate bending moment
*b, t*	dimensions of the rectangular cross-section
*t*_1_, *t*_2_	coordinates describing the locations of rebars
*x*	coordinate describing the location of the neutral axis
*σ_s_*_1_	compressive stress of steel
*σ_s_*_2_	tensile stress of steel
*μ*_1_, *μ*_2_	reinforcement ratios of steels in compression and in tension (rectangle)
*μ*	reinforcement ratio (ring)
*R*	outer radius
*r*	inner radius
*t* = *R* − *r*	thickness of ring
*r_m_*	mean radius
*r_s_*	radius describing the locations of reinforcing steel on the reference circumference
*d_m_*	mean diameter
*α*	angle describing the location of the neutral axis, rad
*ρ* coefficient	*ρ* = *r_s_*/*r_m_*
*ρ_R_* coefficient	*ρ_R_* = *R*/*r_m_*
$\varepsilon^{\prime }$	maximum compressive strain in concrete, ‰
$\varepsilon_{\alpha }^{\prime }=\varepsilon^{\prime }/(\rho_{R}-\cos\alpha )$ *;*	
*ϕ*	angular coordinate, rad
*dA_s_, dA_c_*	element of the steel area *A_s_* and of the concrete area *A_c_*, respectively
*F_a_*_1_, *F_a_*_2_	areas of reinforcing steels in compression and in tension, respectively
